# *QuickStats*: Percentage of Emergency Department Visits That Had an Opioid* Ordered or Prescribed, by Age Group — National Hospital Ambulatory Medical Care Survey, United States, 2006–2015^^†^^

**DOI:** 10.15585/mmwr.mm6711a8

**Published:** 2018-03-23

**Figure:**
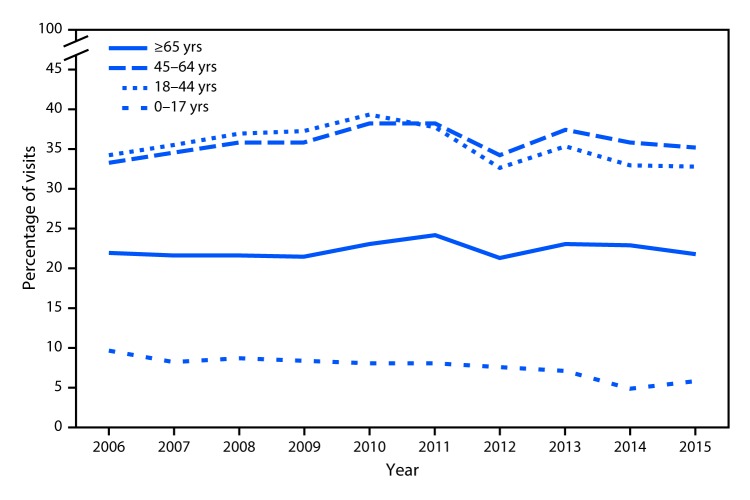
During 2006–2010, the percentage of emergency department (ED) visits that had an opioid ordered or prescribed increased among visits involving persons aged 18–44 years (from 34.3% to 39.3%) and 45–64 years (from 33.2% to 38.3%). However, during 2010–2015, the percentage decreased among visits for those aged 18–44 years (32.7% in 2015) and 45–64 years (35.2% in 2015). Throughout 2006–2015, the percentage decreased among visits for persons aged 0–17 years (from 9.5% in 2006 to 5.7% in 2015), remained stable among visits for those aged ≥65 years, and was highest among visits for those aged 18–44 and 45–64 years.

**Source:** National Center for Health Statistics. National Hospital Ambulatory Medical Care Survey, 2006–2015. https://www.cdc.gov/nchs/ahcd/ahcd_questionnaires.htm.

For more information on this topic, CDC recommends the following link: https://www.cdc.gov/drugoverdose/prescribing/guideline.html.

